# Concurrent Infection with SARS-CoV-2 and *Orientia tsutsugamushi* during the COVID-19 Pandemic in the Maldives

**DOI:** 10.3390/tropicalmed8020082

**Published:** 2023-01-25

**Authors:** Rajib Kumar Dey, Hisham Ahmed Imad, Pyae Linn Aung, Mohamed Faisham, Muaz Moosa, Mariyam Hasna, Aminath Afaa, Thundon Ngamprasertchai, Wasin Matsee, Wang Nguitragool, Emi E. Nakayama, Tatsuo Shioda

**Affiliations:** 1 Department of Medicine, Indira Gandhi Memorial Hospital, Malé 20002, Maldives; 2 Mahidol Vivax Research Unit, Faculty of Tropical Medicine, Mahidol University, Bangkok 10400, Thailand; 3 Thai Travel Clinic, Hospital for Tropical Diseases, Bangkok 10400, Thailand; 4 Center for Infectious Disease Education and Research, Department of Viral Infections, Research Institute for Microbial Diseases, Osaka University, Osaka 565-0871, Japan; 5 Department of Clinical Tropical Medicine, Faculty of Tropical Medicine, Mahidol University, Bangkok 10400, Thailand; 6 Department of Molecular Tropical Medicine and Genetics, Faculty of Tropical Medicine, Mahidol University, Bangkok 10400, Thailand

**Keywords:** scrub typhus, *Orientia tsutsugamushi*, COVID-19, SARS-CoV-2, co-infection, ARDS

## Abstract

The COVID-19 pandemic was the worst public-health crisis in recent history. The impact of the pandemic in tropical regions was further complicated by other endemic tropical diseases, which can cause concurrent infections along with COVID-19. Here, we describe the clinical course of a patient with concurrent COVID-19 and scrub typhus infection. The patient’s de-identified clinical data were retrieved retrospectively. The patient had progressive breathlessness at the time of presentation and was hospitalized for COVID-19. Respiratory examination revealed dyspnea, tachypnea, and coarse crepitations bilaterally over the entire lung field. Oxygenation was impaired, and a PaO_2_/FiO_2_ ratio of 229 suggested acute respiratory distress syndrome. Laboratory tests indicated leukocytosis, thrombocytopenia, ferritinemia, hypoalbuminemia, and transaminitis. Upon revaluation for persistent fever, physical examination revealed an eschar in the right antecubital fossa. Serology further confirmed scrub typhus, with IgM and IgG antibody positivity. A remarkable clinical recovery was achieved with doxycycline. The COVID-19 pandemic might have masked endemic tropical diseases. Clinicians working in endemic regions must always consider common tropical diseases that may present as a co-infection, as in our case. Travel and exposure history are critical guides for narrowing down a differential diagnosis. Early diagnosis and treatment can prevent complications.

## 1. Introduction

The unprecedented pandemic, which unfolded in 2020, was one of the worst global crises in recent history [1]. The cause was identified as a coronavirus from the first cluster of human cases who developed pneumonia in December 2019 after exposure to the China Huanan seafood market [2]. Although the origins of the novel coronavirus are still unclear, the identified virus was subsequently defined as severe acute respiratory syndrome associated coronavirus 2 (SARS-CoV-2), and the disease is referred to as coronavirus disease 2019 (COVID-19) [3].

SARS-CoV-2 is a highly transmissible ribonucleic acid (RNA) betacoronavirus, within the family of coronaviridae, and a new zoonotic virus within the sarbecovirus subgenus. This virus, along with the previously identified SARS-CoV-1 and Middle Eastern respiratory syndrome coronavirus (MERS-CoV), belonging to the genus merbecovirus, can cause severe diseases in humans, in contrast to other human alphacoronaviruses, such as HCoV-NL63 and HCoV-229E, and human betacoronaviruses, such as HCoV-OC43 and HCoV-HKU1, which typically cause self-limiting upper respiratory tract infections with a mild clinical course [4]. The spectrum of clinical manifestations of SARS-CoV-2 infection is broad and presently classified as pre-symptomatic, mild to moderate illness, and severe or critical illness. The last includes acute respiratory distress syndrome (ARDS) and multi-organ dysfunction syndrome (MODS), involving a hyper-reactive host inflammatory response, as we have previously reported [5].

The pathogenesis of ARDS and refractory hypoxemia with SARS-CoV-2 is presently uncertain, but it is believed to be principally related to diffuse alveolar damage via multi-factorial pathways, leading to irreversible intracapillary thrombosis [6]. However, the most evident mechanism triggering ARDS is cytokine storm activation of the endothelium, leading to endotheliitis [7].

Other tropical diseases, such as scrub typhus, also share a common pathogenesis with overwhelming infection, with the development of endotheliitis and ARDS [8]. Scrub typhus is caused by *Orientia tsutsugamushi*, a Gram-negative intra-cytosolic bacterium that belongs to the Rickettsiaceae family, which is transmitted to humans by exposure to infected mites [9]. Until recently, the majority of cases were reported from a geographically restricted region in the eastern hemisphere [10]. Presently, however, it is widely accepted that the geographic distribution of *Orientia* species extends much further, including to South America and Africa [11].

Scrub typhus is an urban disease, and unprotected exposure to mite-infested vegetation or improper handling of rodents have been implicated in its development [12]. These tiny red mites measure 1 to 2 mm in size and can be found inside the external ears of rodents, and also in the wing feathers of migratory birds. In endemic areas with a large mite population, the bacterium is maintained through vertical transmission to mite progeny [13,14]. In humans, following exposure and inoculation of the bacterium, the incubation period before symptom development can be as long as three weeks.

The clinical manifestations of scrub typhus begin with a fever with chills and myalgia [15]. Although headache is a predominant feature, scrub typhus’s cardinal feature is an eschar at the inoculation site accompanied by regional lymphadenopathy [12]. This distinct characteristic skin lesion appears as a central necrotizing spherical ulcer with surrounding erythema that is non-tender and not pruritic. The resulting surface scab, which is reported to occur in 60% of cases, appears as a burn wound but is often unnoticed. Apart from this lesion, a transient macular rash can appear over the trunk and extremities, sparing the face and palm. An acute undifferentiated febrile illness is the most common presentation during the early stages of infection. Distinguishing scrub typhus from other diseases caused by co-circulating endemic pathogens is complex [16]. Laboratory confirmation is based on antigen or antibody detection, or other molecular techniques, such as polymerase chain reaction (PCR).

Although laboratory confirmation of scrub typhus is widely unavailable, especially in resource-limited settings, serology remains the primary method for confirmation of the infection in most healthcare or hospital settings. In addition, laboratory features, such as thrombocytopenia and raised liver enzymes against the background of a normal white blood cell count in a febrile patient, are frequent observations reported in previous cohorts [17]. Complications of scrub typhus include pneumonitis, ARDS, acute renal failure, meningoencephalitis, and MODS, occurring more so in cases with delayed diagnosis and treatment. Pneumonitis is the most common complication, which develops around the second week of untreated infection before progressing to ARDS [18,19].

At the time of preparing this manuscript, approximately seven million people have died from COVID-19, and over six hundred million positive cases have been reported worldwide. In the Maldives, there were 185,708 laboratory-confirmed cases (33% of the population) out of which 311 fatalities were recorded since the beginning of the pandemic. Presently, 86% of individuals above the age of 12 years have received at least two doses of vaccine against COVID-19. In addition, we previously reported a case of co-infection of SARS-CoV-2 and dengue virus during the pandemic in the Maldives, which is a tropical region that is endemic for dengue [20]. Here, we present a case of COVID-19 with concurrent scrub typhus in an adult.

## 2. Materials and Methods

The patient was admitted to Indira Gandhi Memorial Hospital (IGMH) in Malé, Republic of Maldives, in January 2022, with chief complaints of fever and breathlessness. De-identified clinical and laboratory data from the patient’s hospitalization were reviewed using in-patient medical charts. The patient had undergone a reverse transcription polymerase chain reaction (rRT-PCR) test for COVID-19 (Liferiver^TM^ 2019-nCoV Real Time Multiplex RT-PCR Kit (San Diego, CA, USA). Diagnostics for *O. tsutsugamushi* included the “Scrub Typhus Detect™” immunoglobulin (Ig)M/IgG enzyme-linked immunosorbent assay (InBios International, Seattle, WA, USA). Dengue diagnostics included SD Bioline Dengue Duo (Abbott Diagnostics, Yonginsi, Gyeonggi, S. Korea) and Panbio Dengue IgM/IgG ELISA (Abbott Diagnostics). Hemoculture was performed using an automated culture system (bioMeriuex Inc., Durham, NC, USA). We also conducted a PubMed search for similar cases using the following MeSH terms: COVID-19, SARS-CoV-2, scrub typhus, *Orientia tsutsugamushi*, and co-infection. Further, we searched for published peer-reviewed case reports on Google Scholar using keywords, such as scrub typhus, COVID-19, co-infection, *Orientia tsutsugamushi*, and SARS-CoV-2. The literature search showed that 22 similar articles have been published since the beginning of the pandemic. After excluding 19 articles that were duplicates, pre-prints, review papers, or papers about co-infection of SARS-CoV-2 with infections other than *Orientia*, we reviewed three articles to compare the clinical findings with those of the present case.

## 3. Case Report

A 40-year-old female presented to the emergency department with complaints of progressive cough and shortness of breath for two days. She looked sick, lethargic, and grossly dyspneic. However, she was completely conscious and coherent at the time of presentation. Initial physical examination revealed a raised body temperature of 38 °C, an increased pulse rate of 108 beats per minute, low blood pressure of 80/50 mmHg, and a rapid respiratory rate of 28 breaths per minute, along with a slightly low oxygen saturation of 92% in room air.

There was no evidence of cyanosis, jaundice, anemia, or signs of severe dehydration. Additionally, there was no obvious cervical or generalized lymphadenopathy, and no findings within the oral cavity suggestive of an infective focus, such as pharyngitis, tonsillitis, or diphtheria. Respiratory examination by auscultation of the chest revealed fine crackles towards the end of inspiration in both lung fields, the intensity of which was not changed by coughing. No other adventitious sounds, such as wheezing, stridor, or pleural rub, were audible during the examination. Further, breath sounds were equal in both lungs, with symmetrical chest movements and a centrally palpable trachea.

Abdominal examination was normal, with a soft, non-tender abdomen without evidence of hepatosplenomegaly, except for the involuntary use of accessory muscles to aid respiration. No complaints of abnormal bowel movement, diarrhea, or urinary symptoms were reported.

Cardiovascular examination revealed tachycardia with normal heart sounds, while the neurological findings were unremarkable, with no neck stiffness or apparent neurological deficit. Additional history of the presenting illness included fever with body ache and generalized weakness for the past week. She denied a history of similar illness in the past or testing positive for COVID-19, and had not received any immunization against SARS-CoV-2. Further, her travel history was only significant for travel from her residential atoll to the capital city of Maldives, a distance of approximately 120 km, to consult our hospital (Figure S1).

The requisite pre-admission screening was positive for SARS-CoV-2, as shown in Table 1.

The patient was transferred to an annexed facility reserved for managing COVID-19 cases and was admitted to the intensive care unit with a diagnosis of severe COVID-19. The hypotension at presentation responded to a fluid challenge, and her blood pressure was maintained without the need for additional inotropic support. Arterial blood gas analysis suggested ARDS, with an arterial oxygen partial pressure to fraction of inspired oxygen (PaO2/FiO2) ratio of 229. A portable chest X-ray showed homogenous reticular infiltration with increased infiltration at the peripheries (X-ray not provided). Treatment was commenced with antimicrobials and anti-inflammatory agents, including ceftriaxone 2 g daily, remdesivir 100 mg daily, and dexamethasone 6 mg/day. Additionally, oxygen supplementation and other symptomatic treatment were administered.

**Figure 1:**
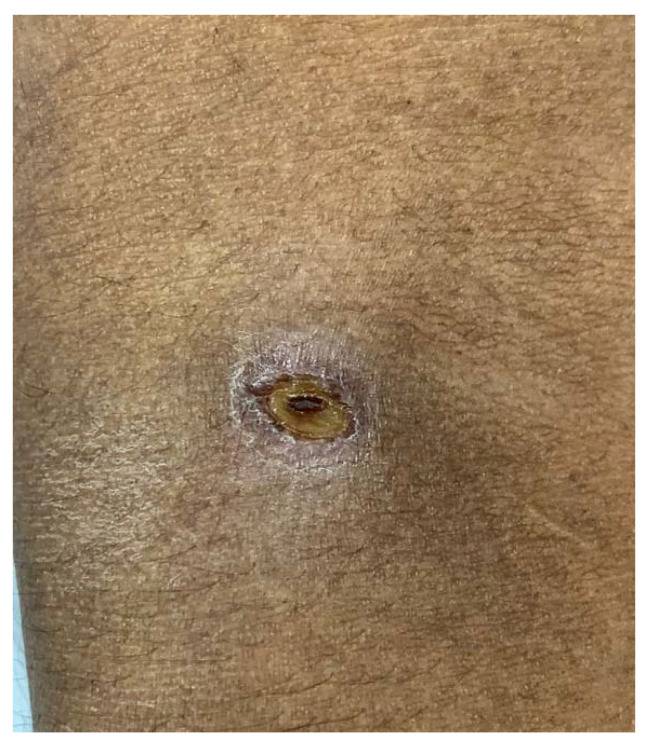
Eschar of scrub typhus (measuring 7 × 5 mm).

However, there was no clinical improvement by the second day of hospitalization. The fever persisted and there was no improvement in dyspnea. Repeat arterial blood gas analysis reflected a compensatory respiratory mechanism, along with transaminitis, hypoalbuminemia, ferritinemia, and lactatemia. This prompted a detailed physical examination, which revealed the presence of an eschar on the right antecubital fossa, as shown in Figure 1.

Doxycycline was added to the treatment regimen on the basis of the eschar, with a loading dose of 200 mg stat followed by 100 mg twice daily. Although the fever subsided on the second day after commencing doxycycline, improvement in the PaO_2_/FiO_2_ ratio was only observed on the fifth day of admission, allowing for discontinuation of supplemental oxygen. The patient subsequently recovered completely and was discharged from the hospital on the tenth day.

## 4. Discussion

Presently, the COVID-19 pandemic is on course to continue for a fourth consecutive year, and several clustered variants have emerged since the beginning of the pandemic [21]. These genomic mutations augment the virus’ infectiousness, making it the most transmissible contagion known. Currently, over 60% of the world’s population is reportedly fully vaccinated against COVID-19, and 70% has received at least one dose. In this population group, re-infection will often manifest as a mild illness, mostly restricted to the upper respiratory tract. However, since the cell surface receptor to which the virus attaches to gain entry into the cell through endocytosis is widely expressed in several organs and tissues within the body, infection can cause a systemic inflammatory process [22]. Hence, infection with SARS-CoV-2 might not only be limited to the respiratory tract, but can also involve other systems, such as the gastrointestinal or genitourinary systems. With growing evidence suggesting waning immunity to SARS-CoV-2, a booster dose of the vaccine is currently being provided to individuals for whom the interval since their last vaccination dose is four months or longer [23]. This is considered a precautionary measure for the anticipated surge in the number of cases during the winter period.

Several tropical diseases are endemic in the Maldives, including scrub typhus [16,24,25,26,27,28]. We previously reported on the epidemiology of scrub typhus in the Maldives and identified certain atolls considered as hotspots for scrub typhus within the archipelago [15]. Additionally, sporadic cases have been previously reported from the Lhaviyani Atoll where our patient resides. However, we could not precisely identify any activities of our patient that might have exposed her to infected mites. Despite this, it is evident that many inhabitants in the atolls who are in the informal sector, including small agricultural holders, are consistently exposed to mites and are prone to infection or carrying back the mites to their homes.

We postulate that our patient might have been infected with *O. tsutsugamushi* at some point in the three weeks prior to the development of her first symptom, and that she acquired SARS-CoV-2 after developing these symptoms but prior to presenting to the emergency department with respiratory distress. Complications, such as ARDS, in scrub typhus usually develop after the second week of infection without treatment, while in COVID-19, ARDS usually develops after day seven of infection. In the presented case, ARDS developed quite acutely after the onset of respiratory symptoms. Hence, we speculate that SARS-CoV-2 infection might have aggravated her clinical trajectory, masking the occult infection with *O. tsutsugamushi*.

Based on the initial hematological profile of the presented case, we observed some interesting findings that were typical as well as atypical to both diseases. One of the typical features that correlated with the respiratory symptoms and a positive SARS-CoV-2 RT-PCR test was the presence of lymphopenia, which has previously been reported to occur in COVID-19 [3]. As with other viral illness, SARS-CoV-2 leukopenia is observed during the acute phase, with a right shift in white blood cells. On the other hand, a left shift is usually observed with bacterial infection [29]. In scrub typhus, however, leukocytes remain within the normal range during the acute phase and leukocytosis is only observed during the late stages of severe disease before the individual succumbs to the disease. The finding of leukocytosis with neutrophil predominance at the time of presentation in this case can be considered atypical for a primary diagnosis of COVID-19. Likewise, marked thrombocytopenia is neither a common finding during the early stages of COVID-19 nor a finding of scrub typhus. However, marked thrombocytopenia during the acute phase might be a finding that is observed with co-infections [30].

The exact pathogenesis of thrombocytopenia in both diseases is largely unknown. However, some causes of thrombocytopenia related to COVID-19 have previously been proposed [31]. These include: (i) increased platelet consumption as a result of formation of microthrombi from aggregation of activated platelets due to endothelial injury, (ii) decreased production of megakaryocytes through direct inhibition of bone marrow growth and dyshematopoiesis by the infection, and (iii) increased platelet clearance by auto-antibodies and immune complexes. In scrub typhus, on the other hand, vasculitis is the primary reason for the onset of thrombocytopenia. Additionally, septicemia can deplete circulating platelets in cases of MODS and disseminated intravascular coagulation. Lastly, aggravated host hyper-inflammatory responses can trigger macrophage activation syndrome or hemophagocytic lymphohistiocytosis, where platelets are phagocytized along with other white blood cells.

In our case, both diseases might have contributed to thrombocytopenia. Hence, we advocate clinicians working in endemic regions to consider other causes of infectious thrombocytopenia in patients with COVID-19. In the presented case, her hematological profile at the time of presentation was consistent with the profile observed in leptospirosis, which typically presents with leukocytosis and neutrophilia with thrombocytopenia [32]. However, there was no evidence of renal and hepatic involvement at the time of presentation, which is frequent in hospitalized severe leptospirosis cases. Further, leptospirosis is considered to be sporadic in the Maldives and our patient had no history of exposure to rodents or flood water that could have led to contact with leptospira.

Discovering the eschar was key to making the clinical diagnosis of scrub typhus in our case. Eschars are important and valuable clinical findings that can aid the clinical diagnosis of scrub typhus, allowing for the initiation of prompt treatment, since a delay in treatment can have devastating consequences. Eschars are typically located on unexposed areas of the body, and the distribution of eschars has been previously described [33]. Additionally, the eschar morphology might vary in size or appearance based on the infecting serotype or following re-infection [34]. Atypical eschars, appearing as ulcers without central necrosis, have also been previously described [35,36]. In the present case, the eschar appeared to have the features of an ulcer, but also had other characteristic features of an eschar, such as central necrosis with surrounding erythema and a collar of desquamation. Apart from facilitating a visual diagnosis, and since *O. tsutsugamushi* DNA can be detected in the eschar by PCR, it can be used for molecular confirmation, even after initiation of treatment [37,38]. In fact, we previously described laboratory confirmation of *O. tsutsugamushi* via molecular analysis using the dried scab of the eschar [12].

We compared the present case with similar cases of co-infection with SARS-CoV-2 and *O. tsutsugamushi* previously reported in the literature, including the available signs and symptoms, laboratory findings, treatment, and outcomes (Table S1) [39,40,41]. The previous cases included two females and two males, and consisted of one child and three adults. All cases were from the Indian subcontinent, since this region is endemic for scrub typhus. Fever was reported in all four cases: a high-grade fever is usually associated with scrub typhus in more than 90% of patients at the time of admission [42]. Although viral infections usually manifest with the abrupt onset of high-grade fever, most patients with COVID-19 present with low-grade fever or chills [43]. Our experience suggests that fever seems to be a prominent feature of systemic illnesses and, when it is persistent, clinicians should broaden the differential diagnosis and consider including common tropical diseases. Unfortunately, however, the systemic symptoms are non-specific and might overlap in both diseases or exhibit atypicality [44]. For example, our case did not manifest any typhus features of confusion, which is a cardinal feature of scrub typhus and other rickettsioses [45]. The other hallmark feature of scrub typhus, the eschar, was only discovered in two of the five cases reviewed. Thrombocytopenia and transaminitis are additional findings common to scrub typhus that were also observed in cases with co-infection of SARS-CoV-2 and *O. tsutsugamushi.* These two hematological and biochemical findings should add weight when narrowing down the differential diagnosis to scrub typhus.

In the absence of an eschar in patients presenting with an acute undifferentiated febrile illness, scrub typhus must be primarily considered if there is an epidemiological link to an endemic region. In addition, during acute infection, when laboratory investigations reveal a normal leukocyte count with thrombocytopenia and transaminitis, and if all likely viral etiologies are excluded, we recommend empiric treatment with doxycycline, provided there are no contraindications, such as pregnancy or prior documented allergies to tetracycline antibiotics. Furthermore, doxycycline should only be used for life-threatening conditions in children under the age of eight years. Further, the presence of hepatosplenomegaly would support a diagnosis of rickettsioses. In scrub typhus, hepato-splenomegaly occurs as a result of vasculitis, which is the cardinal pathophysiology in scrub typhus. Further, during rickettsemia, a transient evanescent maculopapular rash is present, which can be an additional finding supporting the diagnosis of rickettsial infection. Additionally, we recommend performing serological investigations using an indirect immunofluorescence assay, which is the gold-standard test for the diagnosis of scrub typhus.

This report has several limitations due to its retrospective nature. First, the patient’s medical chart, including X-ray, was not available as both were segregated as part of measures to mitigate SARS-CoV-2 transmission. Hence, we could only obtain electronic data. Further, there were no stored blood specimens or eschar to perform further molecular analysis, such as determining the infection serotype.

The COVID-19 pandemic might have masked several endemic tropical diseases. Clinicians working in endemic regions must always consider common tropical diseases that might present as co-infections, as in our case. Travel and exposure history are critical factors that can help narrow down the differential diagnosis. Early recognition and treatment of scrub typhus infection can prevent complications.

## Figures and Tables

**Table 1:** Serial laboratory parameters from the time of presentation to the end of follow up.

Day of Illness (Days)	Day 7	Day 8	Day 10	Day 15
Hospitalization (Days)	Day 1	Day 2	Day 4	Follow Up
Leukocytes (5000–10,000/µL)	12,800		12,200	9400
Neutrophils (40–60%)	85.0		72.0	72.0
Lymphocytes (12.2–47.1%)	9.0		23.0	22.0
Eosinophils (0.0–4.4%)	0.0		0.0	0.0
Basophils (0.0–0.7%)	0.0		0.0	0.0
Monocytes (4.4–12.3%)	6.0		5.0	6.0
Hemoglobin (11.9–15.4 g/dL)	12.0		12.2	12.6
Hematocrit (36.2–46.3%)	36.6		36.8	37.0
Platelets (151,000–304,000/µL)	75,000		123,000	176,000
Creatinine (0.7–1.2 mg/dL)	0.68		0.70	0.70
Urea (19.0–44.1 mg/dL)	8.0		10.0	10.0
Sodium (136–145 mmol/L)	135		137	
Potassium (3.5–5.1 mmol/L)	3.8		3.7	
Total Bilirubin (0.2–1.2 mg/dL)		2.9	1.3	0.7
Direct Bilirubin (0.0–0.5 mg/dL)		2.0	0.9	0.3
Albumin (35–5.2 g/dL)		2.4	2.8	3.2
Protein (6.4–8.3 g/dL)		5.3	5.8	6.2
Aspartate aminotransferase 5.0–34.0 IU/L)		156	93	35
Alanine aminotransferase (0.0–55.0 IU/L)		95	63	47
Alkaline phosphatase (40.0–150.0 IU/L)		347	270	156
Prothrombin time (11–13.5 s)		12/1.0		
aPTT (30–40 s)		35		
d-Dimer (≤0.50 mg/L FEU)		13.55	4.56	
CRP (0.0–0.5 mg/dL)		26.0	13.8	2.3
LDH (140–280 IU/L)		726	510	256
Lactate (0.0–1.0 mmol/L)		3.6		
Ferritin (10–120 ng/mL)		4887	4165	
Hepatitis B surface Ag		Negative		
Anti-hepatitis B surface Ab		Negative		
Anti-hepatitis C Ab		Negative		
Anti-HIV Ag/Ab		Negative		
Dengue NS1		Negative		
Anti-dengue IgM		Negative		
Anti-dengue IgG		Negative		
SARS-CoV-2 RT-PCR		Positive		
Scrub typhus IgM		Positive		
Scrub typhus IgG		Positive		
Blood culture		No growth		
Sputum culture		No growth		

aPTT: activated partial thromboplastin time; FEU: fibrinogen equivalent units; d-Dimer: domain dimer; CRP: c-reactive protein; LDH: lactate dehydrogenase; Ag: antigen; Ab: antibody; IgM: immunoglobulin M; IgG: immunoglobulin G; SARS-CoV-2: severe acute respiratory syndrome coronavirus 2; RT-PCR: real-time polymerase chain reaction.

## Data Availability

The data presented in this study are available on request from the corresponding author. The data are not publicly available to ensure the privacy of the study participant.

## Supplementary Materials

The following supporting information can be downloaded at: https://www.mdpi.com/article/10.3390/tropicalmed8020082/s1, Figure S1: Map of Maldives; Table S1: Summary of cases co-infected with SARS-CoV-2 and *Orientia tsutsugamushi.*
