# Supplementary material for: Concurrent Infection with SARS-CoV-2 and Orientia tsutsugamushi during the COVID-19 Pandemic in the Maldives
Source: Trop Med Infect Dis. 2023 Jan 25;8(2):82. doi: 10.3390/tropicalmed8020082 (PMC9959419; doi:10.3390/tropicalmed8020082)
Supplement: Supplementary file 1 [file tropicalmed-08-00082-s001.zip › tropicalmed-2161430-supplementary.pdf]

Supplementary Materials of Concurrent Infection with SARS-CoV-2 and *Orientia tsutsugamushi* during the COVID-19 Pandemic in the Maldives

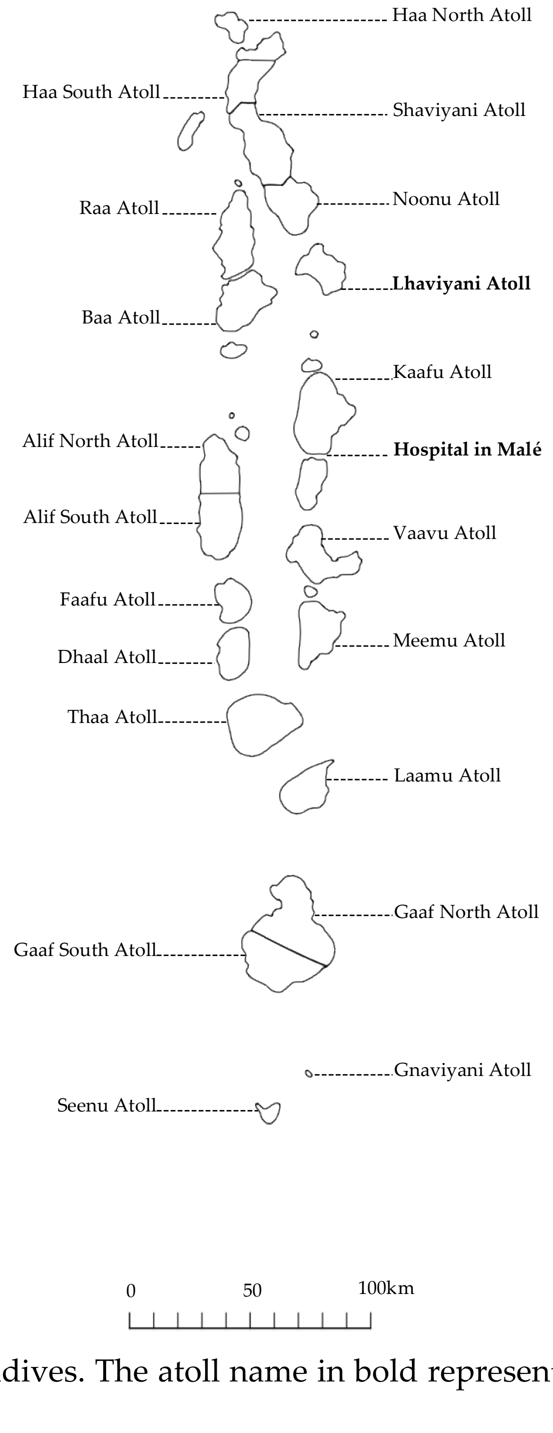

Figure S1. Map of Maldives. The atoll name in bold represents the residence atoll of the presented case.

Table S1. Summary of cases in the literature and the present case that were co-infected with SARS-CoV-2 and *Orientia tsutsugamushi*.

Clinical Findings	Present Case	Other Cases			
		Case I[39]	Case II[40]	Case III[41]	Case IV[41]
Patient's characteristics	40-year-old female from Maldives	14-year-old female from Nepal	▪ 70-year-old female from south India	▪ 55-year-old male from central India	▪ 35-year-old male from north central India
Presenting complaints, symptoms and signs of illness	▪ Fever<br>▪ Body aches<br>▪ Fatigue<br>▪ Cough<br>▪ Dyspnea	▪ Fever<br>▪ Headache<br>▪ Myalgia<br>▪ Arthralgia<br>▪ Cough<br>▪ Dyspnea	▪ Fever<br>▪ Fatigue<br>▪ Myalgia	▪ Fever<br>▪ Cough<br>▪ Dyspnea<br>▪ Altered sensorium	▪ Fever<br>▪ Cough<br>▪ Dyspnea<br>▪ Altered sensorium
Onset of symptoms prior to presentation	▪ 10 days	▪ 7 days	▪ 3 days	▪ unrecorded	▪ 7 days
Vital signs and finding supporting of clinical diagnosis	▪ Temperature: 38.0°C<br>▪ BP: 80/50mmHg<br>▪ PR: 108 beats per min<br>▪ RR: 28 breaths per min<br>▪ SPO_2: 92% (room air)<br>▪ Eschar: on the right antecubital fossa<br>▪ Crackles audible on auscultation of both lung fields	▪ Temperature: 38.3°C<br>▪ BP: 108/72 mmHg<br>▪ PR: 112 beats per min<br>▪ RR:<br>▪ SPO_2: 95% (room air)<br>▪ Eschar: unreported	▪ Temperature:<br>▪ BP: 120/80mmHg<br>▪ PR: 80 beats per min.<br>▪ RR: 20 breaths per min.<br>▪ SpO_2: 99% (room air)<br>▪ Eschar: on the left infra-axillary region	▪ Temperature: unrecorded<br>▪ BP: 100/58 mmHg<br>▪ PR: 54 beats per min.<br>▪ RR: 20 breaths per min.<br>▪ SPO_2: 100% (room air)<br>▪ Eschar: not found	▪ Temperature: unrecorded<br>▪ BP: 130/90 mmHg<br>▪ PR: 140 beats per min.<br>▪ RR: 38 breaths per min.<br>▪ SPO_2: 50% (room air)<br>▪ Eschar: not found
Hematological and biochemical abnormalities and findings on imaging.	▪ Thrombocytopenia<br>▪ Transaminitis<br>▪ Leukocytosis<br>▪ Lymphopenia<br>▪ Hypoalbuminemia<br>▪ Ferritinemia<br>▪ Chest x-ray: interstitial involvement	▪ Thrombocytopenia<br>▪ Transaminitis<br>▪ Leukocytopenia<br>▪ Chest x-ray and computer tomography: dextrocardia	▪ Thrombocytopenia<br>Leukocytosis	No thrombocytopenia<br>Transaminitis<br>Leukocytosis<br>▪ Lymphopenia<br>▪ Hypoalbuminemia<br>▪ Elevated IL-6<br>▪ Ferrintinemia	No thrombocytopenia<br>▪ Transaminitis<br>▪ Leukocytosis<br>▪<br>▪ Hypoalbuminemia
Laboratory confirmation: scrub typhus/ COVID-19	▪ SARS-CoV-2 RT-PCR: +ve<br>▪ Scrub typhus IgM/IgG antibodies: +ve	▪ SARS-CoV-2 RT-PCR: +ve<br>▪ Scrub typhus IgM/IgG antibodies: +ve	▪ SARS-CoV-2 RT-PCR: +ve<br>▪ Scrub typhus qPCR: +ve	▪ SARS-CoV-2 RT-PCR: +ve<br>▪ Scrub typhus IgM ELISA: +ve	▪ RT-PCR for COVID-19: + ve<br>▪ IgM ELISA: +ve
Complications	▪ ARDS	▪ Unreported	▪ Unreported	▪ Meningoencephalitis	▪ ARDS and MODS
Antimicrobial therapy	▪ Ceftriaxone<br>▪ Remdesivir<br>▪ Doxycycline	▪ Unreported	▪ Doxycycline	▪ Ceftriaxone<br>▪ Vancomycin<br>▪ Doxycycline	▪ Hydroxychloroquine<br>▪ Piperacillin/tazobactam<br>▪ Meropenem<br>▪ Doxycycline
Outcomes	▪ Recovered	▪ Unreported	▪ Recovered	▪ Recovered	▪ Fatal

BP: blood pressure, PR: pulse rate, RR: respiratory rate, SpO_2 : oxygen saturation, IL-6: interleukin 6, SARS-CoV-2; severe acute respiratory syndrome associated virus 2, RT-PCR; reverse transcriptase polymerase chain reaction, IgM: immunoglobulin M, IgG: immunoglobulin G, ELISA: enzyme linked immunosorbent assay, COVID-19; coronavirus disease 2019.
